# Impact of BioFire FilmArray respiratory panel results on antibiotic days of therapy in different clinical settings

**DOI:** 10.1017/ash.2021.164

**Published:** 2021-06-24

**Authors:** Jenna J. Manatrey-Lancaster, Amanda M. Bushman, Meagan E. Caligiuri, Rossana Rosa

**Affiliations:** 1 Department of Pharmacy, UnityPoint Health-Des Moines, Des Moines, Iowa; 2 Infectious Diseases Service, UnityPoint Health-Des Moines, Des Moines, Iowa; 3 Department of Internal Medicine, University of Iowa-Des Moines Campus, Des Moines, Iowa

## Abstract

**Objective:**

The BioFire FilmArray Respiratory Panel (RFA) has been proposed as a tool that can aid in the timely diagnosis and treatment of respiratory tract infections but its effect on antibiotic prescribing among adult patients has varied. We evaluated the impact of RFA result on antibiotic days of therapy (DOTs) in 2 distinct cohorts: hospitalized patients and patients discharged from the emergency department (ED).

**Design:**

Retrospective cohort study.

**Setting:**

The study was conducted in 3 community hospitals in Des Moines, Iowa, from March 3 to March 16, 2019.

**Patients:**

Adults aged >18 years.

**Methods:**

Potential outcome means and average treatment effects for RFA results on antibiotic DOTs were estimated. Inverse probability of treatment weighting with regression adjustment was used.

**Results:**

We identified 243 patients each in the hospitalized and ED-discharged cohorts. Among hospitalized patients, RFA results did not affect antibiotic DOTs. Among patients discharged from the ED, we found that if all patients had had influenza detected, the average DOTs would have been 2.3 DOTs (−3.2 to −1.4) less than the average observed if all the patients had had a negative RFA (*P* < .0001); no differences in DOTs were observed when comparing an RFA with a noninfluenza virus detected compared to an RFA with negative results.

**Conclusions:**

The impact of RFA results on antibiotic DOTs varies by clinical setting, and reductions were observed only among patients discharged from the ED who had influenza A or B detected.

Respiratory tract infections are a leading cause of outpatient medical and emergency department visits, hospitalizations, and antimicrobial prescriptions across all age groups.^1,2^ Although most respiratory infections are caused by viruses, it is difficult to differentiate between viral or bacterial infections based on clinical signs and symptoms alone.^3^ It can also be difficult for clinicians to differentiate between uncomplicated and complicated viral respiratory infections, bacterial pneumonia, and secondary bacterial infections using traditional microbiological studies alone.^4^ This clinical uncertainty is thought to explain the overuse of antibiotics and other diagnostic tests such as chest radiographs, procalcitonin (PCT), and C-reactive protein (CRP) levels, which can contribute to increases in overall healthcare costs.^1,5^ Moreover, an estimated ∼41 million unnecessary antibiotic prescriptions are written annually in the United States for viral respiratory infections, which amounts to ∼US$1.1 billion in unnecessary healthcare spending.^3^ In addition to direct cost, excess use of antibiotics have additional implications including antibiotic resistance as well as adverse events such as allergic reactions, side effects, and *Clostridiodes difficile* infections.^6–9^

Accurate and timely diagnosis of respiratory tract infections has been proposed as a tool to limit inappropriate antibiotic use, reduce costs, and improve quality of care.^10,11^ Molecular diagnostics like the BioFire FilmArray respiratory panel (RFA, bioMèrieux, Marcy-l’Étoile, France) can provide rapid pathogen identification that can aid in the diagnosis of viral and bacterial respiratory tract infections. The RFA has a high sensitivity and specificity, quick turnaround time, and high diagnostic yield. Compared to traditional bacterial and viral culture methods, which can take days to finalize, the RFA provides results in about an hour with as little as 10 minutes of hands-on time.^12,13^ The RFA also has a diagnostic yield of up to 90% compared to traditional sputum cultures, which have a diagnostic yield below 50%.^13,14^ Enzyme immunoassays may be used for the detection of influenza and respiratory syncytial virus, but their results are limited due to their low sensitivity.^15,16^ The advantages of the RFA have been proposed to facilitate antimicrobial stewardship and to lower healthcare costs, but studies in adult patients have yet to demonstrate a consistent impact on clinical outcomes and costs. We evaluated the impact of RFA results on antibiotic days of therapy (DOTs) in 2 distinct cohorts, hospitalized patients and patients discharged from the emergency department (ED).

## Methods

### Design, outcome and primary exposure

In this retrospective cohort study, we collected data from the records of adult patients presenting to any of 3 teaching hospitals that are part of an integrated health system in Des Moines, Iowa. The main outcome of interest was antibiotic DOTs and the main exposure of interest was RFA result.

Antibiotic DOT was defined as the aggregate sum of days any antimicrobial agent was administered or prescribed to a patient as documented in the electronic medical record (EMR). RFA result was categorized in 3 levels: influenza (if influenza A or B were detected), noninfluenza virus (if any other viral target was detected), and negative (if no targets were detected).

### Data collection procedures and definitions

Eligible patients were identified from the microbiology database. We included patients aged >18 years who had an RFA test within 48 hours of presentation to the emergency department or hospital admission during the 2 weeks of peak influenza cases in Iowa during 2019, spanning March 3 to March 16. To capture real-world practice patterns, we purposefully included any patient who had an RFA ordered, not just those who were ultimately diagnosed as having a respiratory infection. Patients with a definitive indication for antibiotic use (ie, bacteremia, osteomyelitis, and endocarditis), regardless of RFA result, and those with bacteria detected on RFA were excluded. Patients were divided into 2 cohorts according to their disposition status and classified as hospitalized or discharged from the ED.

The following data were collected: demographic information, syndrome upon presentation, RFA result, bacterial culture results, antibiotic(s) received, duration of antibiotic therapy, chest radiograph results, PCT, and white blood cell (WBC) counts if obtained, initial vital signs, comorbid conditions (eg, diabetes mellitus, congestive heart failure, cancer, history of transplant or currently receiving dialysis), and admission status (ED-discharged versus hospitalized). Syndrome on presentation was collected from the RFA ordering clinician’s progress note and categorized as pneumonia, bronchitis, influenza or influenza-like illness, sepsis, nonrespiratory infection, noninfectious process, or acute hypoxic respiratory failure. Chest radiographs were categorized as not obtained, clear or noninfectious, concerning for infection, or indeterminate based on radiologist’s interpretation. Fever was defined as a temperature >38°C; WBC considered low if <4.0×10^3^/µL and elevated if >12.0 × 10^3^/µL, and PCT was considered low if <0.05 ng/mL and elevated if >0.25 ng/mL.

### Statistical analysis

Continuous variables were described using median and interquartile range (IQR) and categorical variables were described using proportions. The Kruskal-Wallis test and χ^2^ test were applied as appropriate.

A potential outcomes model was applied. Briefly, this model specified the potential outcomes that each individual would obtain under each treatment level. We then estimated the potential outcome means (the average potential outcome for each treatment level) and the average treatment effect (the average effect of the treatment in the population).^17^ For the purposes of our study, the potential outcome means was the average antibiotic DOTs for each RFA result level and the average treatment effect was the average effect on antibiotic DOTs if every patient in the cohort had had a negative RFA result versus a noninfluenza virus, and a negative result versus an influenza result. Inverse probability of treatment weighting with regression adjustment was used, modeling the exposure (RFA result) using logit regression and the outcome (antibiotic DOTs) with Poisson regression. Covariate selection was based on prior knowledge of their clinical significance for each of the cohorts. Among hospitalized patients, the exposure model included age and fever, and the outcome model included age, fever, WBC result, and urine-culture order. Among patients discharged from the ED, the exposure model included age and fever and the outcome model included age, fever and urine culture order. Diagnostic checks were performed by plotting the estimated densities of the probability of getting each exposure level, and covariate balance was assessed by calculating the standardized differences and variances. All analyses were conducted using Stata version 13 software (StataCorp, College Station, TX).

### Ethics

The study was approved by and conducted in accordance with the UnityPoint Health–Des Moines Institutional Review Board.

## Results

In total, 513 patients had an RFA obtained during the study period. We excluded 27 patients: 20 due to bacteremia and 7 due to documented infection requiring prolonged antibiotic therapy. Finally, 486 patients were included, with 243 in each the hospitalized and ED-discharged cohorts. Patients who were hospitalized were older compared to those who were discharged from the ED, with a median age of 66 years (interquartile range [IQR], 56–79) versus 48 years (IQR, 28–63) respectively, and they had a higher proportion of comorbid conditions, except for organ transplant.

### Hospitalized patients

Among hospitalized patients, 148 (60.9%) had a negative RFA result; 58 (23.9%) had influenza detected and 36 (14.8%) had a noninfluenza virus detected. The baseline demographic characteristics of this group are presented in Table 1. Age and sex distribution were similar among patients in the different exposure groups. Comorbid conditions also occurred in similar proportions, except for dialysis, which was more frequent among patients with noninfluenza virus detected on RFA. Patients with influenza detected on RFA had higher proportions of fever upon presentation (36.2%), chest radiograph result concerning for infection (25.9%), and normal WBC count (77.6%) compared to those with noninfluenza virus and negative RFA results.

**Table 1.** Baseline Demographic Characteristics by BioFire FilmArray Respiratory Panel Result According to Clinical Setting

Hospitalized Patients				
Variable	Negative (N=148), No. (%)	Noninfluenza Virus (N=37), No. (%)	Influenza (N=58), No. (%)	*P* Value
Age (median, IQR)	68.5 (54.5–78)	70 (56–81)	71.5 (60–81)	.21
Sex, male	65 (43.9)	17 (46.0)	27 (46.6)	.93
**Ordering service**				.52
Emergency department	116 (78.4)	30 (81.1)	51 (87.9)	
Internal medicine	16 (10.8)	4 (10.8)	4 (6.9)	
Critical care	8 (5.4)	0	1 (1.7)	
Other	8 (5.4)	3 (8.1)	2 (3.4)	
**Comorbid conditions**				
Diabetes mellitus	51 (34.5)	14 (37.8)	21 (36.2)	.92
CHF	40 (27.0)	8 (21.6)	11 (19.0)	.44
Cancer	19 (12.8)	3 (8.1)	4 (6.9)	.40
Solid-organ transplant	2 (1.4)	1 (2.7)	0	.50
Dialysis	3 (2.0)	5 (13.5)	0	.001
Urine culture obtained	60 (40.5)	21 (56.8)	28 (48.3)	.17
**Chest radiograph result**				.05
Not obtained	23 (15.5)	7 (18.9)	0	
Clear	50 (33.8)	13 (35.1)	19 (32.8)	
Concerning for infection	27 (18.2)	7 (18.9)	15 (25.9)	
Indeterminate	48 (32.4)	10 (27.0)	24 (41.4)	
Fever^a^	23 (15.5)	4 (10.8)	21 (36.2)	.001
**WBC results^b^**				.07
Normal	87 (58.8)	25 (67.6)	45 (77.6)	
Elevated	56 (37.8)	10 (27.0)	10 (17.2)	
Low	5 (3.4)	2 (5.4)	3 (5.2)	
**Procalcitonin level^c^**				.01
Low	37 (25)	8 (21.6)	28 (48.3)	
Elevated	23 (15.5)	8 (21.6)	5 (8.6)	

Patients Discharged From the Emergency Department				
Variable	Negative (N=110), No. (%)	Noninfluenza Virus (N=34), No. (%)	Influenza (N=99), No. (%)	*P* Value
Age, median y (IQR)	50 (33–63)	45.5 (23–65)	41 (25–61)	.14
Sex, male	45 (40.9)	13 (38.2)	36 (36.4)	.80
**Ordering service**			0.42	
Emergency department	108 (98.2)	34 (100)	98 (99.0)	
Internal medicine	2 (1.8)	0	0	
Other	0	0	1 (1.0)	
**Comorbid conditions**				
Diabetes mellitus	19 (17.3)	4 (11.8)	13 (13.1)	.61
CHF	9 (8.2)	2 (5.9)	3 (3.0)	.28
Cancer	4 (3.6)	1 (2.9)	1 (1.0)	.47
Solid-organ transplant	2 (1.8)	1 (2.9)	3 (3.0)	.84
Dialysis	1 (0.9)	0	0	.54
Urine culture obtained	22 (20)	4 (11.8)	18 (18.2)	.55
**Chest radiograph result**				.63
Not obtained	41 (37.3)	11 (32.3)	36 (36.4)	
Clear	49 (44.6)	11 (32.3)	41 (41.4)	
Concerning for infection	8 (7.3)	5 (14.7)	7 (7.1)	
Indeterminate	12 (10.9)	6 (17.6)	15 (15.2)	
Fever^a^	15 (13.6)	6 (17.6)	32 (32.3)	0.004
**WBC results^b^**				<.001
Not done	11 (10)	7 (20.6)	22 (22.2)	
Normal	75 (68.2)	25 (73.5)	60 (60.6)	
Elevated	24 (21.8)	1 (2.9)	8 (8.1)	
Low	0	1 (2.9)	9 (9.1)	

Note. IQR, interquartile range; WBC, white blood cell count; CHF, congestive heart failure.

a Fever was defined as a temperature >38°C.

b WBC cutoffs: normal, 4.00–11.00×10^3^/µL; elevated ≥11.00×10^3^/µL; Low **≤**4.00×10^3^/µL.

c Procalcitonin was obtained in only 109 of 243 hospitalized patients. Procalcitonin cutoffs: low <0.05 ng/mL; elevated >0.25 ng/mL.

The syndromic diagnosis on admission and the median antibiotic DOTs according to syndrome are presented in Supplementary Table 1 (online). Patients with influenza result had higher proportion of diagnosis of influenza-like illness (60.3%) than those with noninfluenza virus (2.7%) or negative results (0%), with similar median antibiotic DOTs across all exposure levels. Pneumonia was diagnosed in a higher proportion of patients with a noninfluenza virus (35.1%) than in those with influenza (25.9%) and with a negative RFA (18.9%), and there was no statistically significant difference in the median antibiotic DOTs by RFA result.

The model estimates of the potential outcomes for the mean antibiotic DOTs according to RFA results among hospitalized patients are displayed in Table 2. The mean antibiotic DOTs if all hospitalized patients had had a negative RFA result would have been 3.7 (95% CI, 3.02–4.4); for a noninfluenza virus, it would have been 4.9 DOTs (95% CI, 3.6–6.2); and for influenza it would have been 3.8 DOTs (95% CI, 2.9–4.7). Estimation of the average effect of RFA result on DOTs showed that if all patients had had a noninfluenza virus detected, the average DOTs would have been 1.2 DOTs (95% CI, −0.3 to 2.6) more than the average observed if all patients had had a negative RFA result (*P* = .11). If all patients had had influenza detected, the average DOTs would have been 0.1 DOT (95% CI, −0.1 to 1.2) less than the average observed if all patients had had a negative RFA (*P* = .90).

**Table 2.** Average Antibiotic Days of Therapy and Average Effect of BioFire FilmArray Respiratory Panel Result

Hospitalized Patients			
Variable	Negative	Noninfluenza Virus	Influenza
Average antibiotic days of therapy (IQR)	3.7 (3.02–4.4)	4.9 (3.6–6.2)	3.8 (2.9–4.7)
Average effect of RFA result			
Negative result versus noninfluenza virus	1.2 (−0.3 to 2.6; *P* = .11)		
Negative result versus influenza virus	0.1 (−0.1 to 1.2; *P =* .90)		

Patients Discharged From the Emergency Department			
	Negative	Noninfluenza Virus	Influenza
Average antibiotic days of therapy (IQR)	2.7 (1.9–3.6)	1.8 (0.6–2.9)	0.5 (0.1–0.8)
Average effect of RFA* result			
Negative result versus noninfluenza virus	−0.9 (−2.4 to 0.4; *P =* .18)		
Negative result versus influenza virus	−2.3 (−3.2 to −1.4; *P <* .0001)		

**Note.** IQR, interquartile range; RFA, BioFire FilmArray respiratory panel.

### Patients discharged from the emergency department

Among patients discharged from the ED, 110 (45.3%) had a negative RFA result, 99 (40.7%) had influenza detected and 34 (14.0%) had a noninfluenza virus detected. The baseline demographic characteristics of this group are presented in Table 1. A numerical but not statistically significant difference was noted in age. Patients with an influenza result had a median age of 41 years (IQR, 26–61) compared to those with noninfluenza virus (median age 45.5; IQR, 23–65) and negative RFA result (median age, 50 years; IQR, 33–63). Fever was present in 32.3% of patients with a positive influenza result, compared to only 13.6% of those with a negative RFA result and 17.6% of those with a noninfluenza virus detected (*P* = .004). Patients with a negative RFA result had a higher proportions of elevated WBC counts than those with influenza and noninfluenza virus detected (21.8%, 2.9%, and 8.1%, respectively).

The final syndromic diagnosis in the ED and the median antibiotic DOTs according to syndrome are presented in Supplementary Table 2 (online). Among patients with a positive influenza result, the most common diagnosis was influenza-like illness (93.9%). Among patients with noninfluenza virus, the most common diagnosis was bronchitis (82.3%), and in those with a negative RFA result, it was noninfectious processes (37.3%). Pneumonia was diagnosed in 11.8% of patients with noninfluenza virus, 8.2% of patients with negative influenza results, and 3.03% of patients with positive influenza results. Antibiotic DOTs for this syndrome showed no statistically significant differences across RFA results.

The model estimates of the potential outcomes for the mean antibiotic DOTs according to RFA result among patients discharged from the ED are shown in Table 2. The mean antibiotic DOTs if all patients in this cohort had had a negative RFA result would have been 2.7 (95% CI, 1.9–3.6); for a noninfluenza virus, it would have been 1.8 DOTs (95% CI, 0.6–2.9); and for influenza, it would have been 0.5 DOT (95% CI, 0.1–0.8). Estimation of the average effect of RFA result on DOTs showed that if all patients had had a noninfluenza virus detected, the average DOTs would have been 0.9 DOT (95% CI, −2.4 to 0.4) less than the average observed if all the patients had had a negative RFA results (*P* = .18). If all patients had had influenza detected, the average DOTs would have been 2.3 DOTs (95% CI, −3.2 to −1.4) less than the average observed if all the patients had had a negative RFA (*P* < .0001).

## Discussion

Our results suggest that clinical decision making regarding RFA results is influenced by clinical context (hospitalization versus anticipated discharge) and that medical decision making does not solely rely on rapid molecular target identification.

### Hospitalized patients

Similarly to our results, Qian et al^18^ found that hospitalized patients with an RFA with no targets detected had no differences in mean antimicrobial defined daily doses (DDDs).^18^ Rapid pathogen detection has been thought to potentially contribute to decrease in antibiotic use. To assess this hypothesis, Saarela et al^19^ conducted a randomized controlled trial (RCT) using a polymerase chain reaction (PCR) panel that detected 16 viral targets and evaluated the impact of timing of test result availability (ie, rapid diagnosis [1 day] vs delayed diagnosis [7 days]), and they found no difference in mean antibiotic duration between these 2 groups.^19^ The results by Saarela et al align with those in our study; at the hospitals in our study, the RFA results are usually available within 2–3 hours, yet we found no impact on antibiotic use among hospitalized patients. In a randomized controlled trial, Brendish et al^20^ compared the impact of on-demand versus routine viral respiratory PCR testing among hospitalized patients and found no difference between groups in the overall proportion of patients who received antibiotics or in the mean duration of antibiotics, which, although derived from a different study design, is in line with our results.

In contrast to our findings, an observational study by Keske et al^21^ demonstrated that after introducing RFA testing, there was no decrease in proportions of inappropriate antibiotic use among adult hospitalized patients, but there was a lower mean antibiotic DOTs (9 days before the intervention compared to 6 days after the intervention). Notably, baseline and postintervention DOTs were higher in this study compared to our results. In a randomized controlled trial by Sengchen et al,^22^ patients hospitalized with the diagnosis of pneumonia, acute exacerbation of chronic obstructive pulmonary disease (COPD) or acute exacerbation of bronchiectasis were randomized to an RFA or control (ie, routine PCR for 10 viral pathogens). The main outcome of interest was duration of intravenous antibiotics, which was 7 days in the RFA group and 8 days in the control group (difference, −1.5 days; 95% CI, −2.1 to −0.8 days). Reductions in length of stay, length of antibiotic therapy and hospitalization costs were also reported. Although the overall results of the study were positive, the duration of intravenous antibiotics in both groups was much higher than in our cohort overall, although it was similar to that of those diagnosed with pneumonia. Along with the results by Keske et al,^21^ these findings could suggest limits to the reduction in antibiotic use that can be achieved through molecular diagnostics alone. Gelfer et al^13^ conducted a randomized controlled trial that compared a bundle of common diagnostics plus a laboratory-generated PCR panel versus bundle plus RFA. They found lower DOTs per 1,000 patient days among patients who had virus only detected as part of their work-up in both the bundle plus laboratory-generated and bundle plus RFA groups compared to the bundle-alone group. However, the number of patients that could be evaluated in each study arm was very small due to logistic issues during the study; thus, the inferences that can be gathered from this study are limited. Gilbert et al^23^ conducted a follow-up study enrolling additional patients to the study cohort described by Gelfer et al and confirmed their previous findings although the overall size of the study remained small.

### Patients discharged from the emergency department

In an observational study of outpatients, Green et al^4^ found that patients with influenza detected by RFA had significantly lower antibiotic prescription rates than patients with noninfluenza virus detected or with no pathogens detected, but there were no differences between these last 2 groups. A study by Echeverria et al^14^ did find a decrease in antibiotic prescriptions among patients seen in the ED with symptoms of an acute respiratory infection who had an RFA compared to those who only had an immunofluorescence assay. Interestingly, as part of the protocol of this study, physicians were called with the test results by a study member and were questioned about changes in medical management (antibiotic or antiviral therapy, or complementary studies) between their initial plan and the final management plan once the test results were reported. This action itself could have constituted an intervention if treating physicians felt that the decision to prescribe an antibiotic in cases in which viruses were detected was viewed negatively by study members.

The Infectious Diseases Society of America’s “Guideline for the Implementation of an Antibiotic Stewardship Program” suggests the use of rapid viral testing for respiratory pathogens to reduce the use of inappropriate antibiotics.^24^ However, no recommendation is given regarding the specific viral targets or the number of targets that should be part of a testing panel. Examples of antimicrobial stewardship program–led interventions using rapid molecular viral testing include automatic reporting of respiratory PCR results plus procalcitonin to prompt antibiotic discontinuation^25^ and educational campaigns on interpretation of respiratory PCR results and antibiotics prescribing.^26^ Because our results and those of other researchers indicate that rapid identification of influenza (but not other viruses) impact antibiotic prescribing practices in the ED,^27^ use of less costly panels with a reduced number of targets could be considered in nonimmunocompromised patients. Moreover, deployment of this strategy could be carried out by antimicrobial stewardship programs.

Our study has several limitations. The data reflected a period of high community prevalence of influenza, which could limit the generalizability of our results to noninfluenza seasons. This study was also retrospective in design and relied on clinician’s documentation in the electronic medical record. Inaccuracies in documentation could limit the internal validity of the study. However, the primary objective of the study was to assess how RFA results affected antibiotic DOTs, which was evaluated using data obtained from the medication administration record and microbiological reports, which are less likely to contain inaccuracies. The retrospective nature of the study limited our ability to evaluate the appropriateness of RFA ordering because both ED and hospital admission notes are often filed after a comprehensive work-up has been completed by the treating clinician (including after RFA results are available). Because those results shape what is documented as the final diagnosis, it can be difficult to retrospectively discern whether an RFA was warranted based on a patient’s presenting symptoms. Prospective studies could be conducted to assess the appropriateness of RFA ordering, and results could be utilized to identify an algorithm for RFA ordering. Lastly, this study was conducted prior to the coronavirus disease 2019 (COVID-19) pandemic and the RFA panel utilized in this study did not include severe acute respiratory coronavirus virus 2 (SARS-CoV-2).

In conclusion, RFA results did not affect antibiotic DOTs among hospitalized patients, but among patients discharged from the ED, lower antibiotic DOTs were observed when influenza was detected compared to antibiotics use with a negative RFA result. These findings indicate a need to develop different antimicrobial stewardship strategies to address antibiotic prescribing according to clinical setting. Studies are also needed to identify how the addition of SARS-CoV-2 affects RFA ordering and utilization and a need to re-examine the utility of multiplex respiratory viral panels compared to panels limited to influenza and SARS-CoV-2.

## Supplementary material

For supplementary material accompanying this paper visit https://doi.org/10.1017/ash.2021.164. click here to view supplementary material
