# Supplementary material for: Impact of BioFire FilmArray respiratory panel results on antibiotic days of therapy in different clinical settings
Source: Antimicrob Steward Healthc Epidemiol. 2021 Jun 24;1(1):e4. doi: 10.1017/ash.2021.164 (PMC9495546; doi:10.1017/ash.2021.164)
Supplement: Supplementary file 1 [file S2732494X21001649sup001.docx]

**Supplementary Table 1. Distribution of syndromic diagnosis on admission and antibiotic days of therapy according to BioFire ® FilmArray ® Respiratory Panel result among hospitalized patients**

|  | **Negative**  **N=148 (%)** | **Non-influenza virus**  **N=37 (%)** | **Influenza**  **N=58 (%)** | ***P*-value*** | |
| --- | --- | --- | --- | --- | --- |
| Pneumonia | 28 (18.9) | 13 (35.1) | 15 (25.9) | |  |
| *Days of therapy, median (IQR†)* | 5 (3-9) | 8 (5-8) | 7 (2-8) | | 0.75 |
| Bronchitis | 16 (10.8) | 12 (32.4) | 3 (5.2) | |  |
| *Days of therapy, median (IQR)* | 4 (1-8) | 1 (0-4.5) | 0 (0-2) | | 0.14 |
| Influenza/influenza-like-illness | 0 | 1 (2.7) | 35 (60.3) | |  |
| *Days of therapy, median (IQR)* | - | 4 | 2(0-6) | | 0.81 |
| Sepsis | 11 (7.4) | 1 (2.7) | 0 | |  |
| *Days of therapy, median (IQR)* | 3 (1-10) | 12 | - | | 0.14 |
| Non-Respiratory Infection | 18 (12.2) | 2 (5.4) | 1 (1.7) | |  |
| *Days of therapy, median (IQR)* | 6 (1-8) | 1 (0-2) | 9 | | 0.18 |
| Non-Infectious Process | 65 (43.9) | 6 (16.2) | 4 (6.9) | |  |
| *Days of therapy, median (IQR)* | 0 (0-1) | 0 (0-6) | 2.5(0-6) | | 0.57 |
| Acute Hypoxic Respiratory Failure | 10 (6.8) | 2 (5.4) | 0 | |  |
| *Days of therapy, median (IQR)* | 1 (0-6) | 3 (0-6) | - | | 0.91 |

**P-*value for the Kruskal-Wallis test for difference in median

†IQR: Inter-quartile range

**Supplementary Table 2. Distribution of final syndromic diagnosis and antibiotic days of therapy according to BioFire ® FilmArray ® Respiratory Panel result among patients discharged from the Emergency Department**

|  | **Negative**  **n=110 (%)** | **Non-influenza virus**  **N=34 (%)** | **Influenza**  **N=99 (%)** | ***P*-value*** |
| --- | --- | --- | --- | --- |
| Pneumonia | 9 (8.2) | 4 (11.8) | 3 (3.0) |  |
| *Days of therapy, median (IQR†)* | 6 (6-8) | 5.5 (4-6) | 6 (6-8) | 0.40 |
| Bronchitis | 40 (36.4) | 28 (82.4) | 1 (1.0) |  |
| *Days of therapy, median (IQR)* | 0 | 0 | 0 | 0.76 |
| Influenza/influenza-like-illness | 2 (1.8) | 1 (2.9) | 93 (93.9) |  |
| *Days of therapy, median (IQR)* | 0 | 0 | 0 | 0.94 |
| Sepsis | 1 (0.9) | 0 | 0 |  |
| *Days of therapy, median (IQR)* | 0 | - | - | NA |
| Non-Respiratory Infection | 16 (14.5) | 0 | 1 (1.0) |  |
| *Days of therapy, median (IQR)* | 8 (3.5-11) | - | 1 | 0.29 |
| Non-Infectious Process | 41 (37.3) | 1 (2.9) | 1 (1.0) |  |
| *Days of therapy, median (IQR)* | 0 | 4 | 0 | 0.09 |
| Acute Hypoxic Respiratory Failure | 1 (0.9) | 0 | 0 |  |
| *Days of therapy, median (IQR)* | 0 | - | - | NA |

**P-*value for the Kruskal-Wallis test for difference in median

†IQR: Inter-quartile range
